# Etorphine induces pathophysiology in immobilized white rhinoceros through sympathomimesis that is attenuated by butorphanol

**DOI:** 10.1093/conphys/coaf009

**Published:** 2025-04-04

**Authors:** Jordyn M Boesch, Robin D Gleed, Peter E Buss, Adrian S W Tordiffe, Gareth E Zeiler, Michele A Miller, Francois Viljoen, Brian H Harvey, Stephen A Parry, Leith C R Meyer

**Affiliations:** Department of Clinical Sciences, College of Veterinary Medicine, Cornell University, 930 Campus Road, Ithaca, New York 14853, USA; Department of Clinical Sciences, College of Veterinary Medicine, Cornell University, 930 Campus Road, Ithaca, New York 14853, USA; Veterinary Wildlife Services, South African National Parks, Kruger National Park, Private Bag X402, Skukuza 1350, South Africa; Centre for Veterinary Wildlife Research, Faculty of Veterinary Science, University of Pretoria, Private Bag X04, Onderstepoort 0110, South Africa; Centre for Veterinary Wildlife Research, Faculty of Veterinary Science, University of Pretoria, Private Bag X04, Onderstepoort 0110, South Africa; Department of Paraclinical Sciences, Faculty of Veterinary Science, University of Pretoria, Pathology and Veterinary Public Health Complex, Onderstepoort 0110, South Africa; Department of Companion Animal Clinical Studies, Faculty of Veterinary Science, University of Pretoria, OVAH-building, Onderstepoort Campus, Private Bag X04, Onderstepoort 0110, South Africa; South African Medical Research Council (MRC) Centre for Tuberculosis Research, Division of Molecular Biology and Human Genetics, Faculty of Medicine and Health Sciences, Stellenbosch University, PO Box 241, Cape Town 8000, South Africa; Centre of Excellence for Pharmaceutical Sciences, School of Pharmacy (Division of Pharmacology), North-West University (Potchefstroom Campus), Universiteit Street, Potchefstroom 2531, South Africa; Centre of Excellence for Pharmaceutical Sciences, School of Pharmacy (Division of Pharmacology), North-West University (Potchefstroom Campus), Universiteit Street, Potchefstroom 2531, South Africa; MRC Unit on Risk and Resilience in Mental Disorders, Department of Psychiatry and Mental Health, Neuroscience Institute, Anzio Road, 1st floor, Groote Schuur Hospital, Observatory, Cape Town 7925, South Africa; The Institute for Mental and Physical Health and Clinical Translation, Health and Education Research Building, Barwon Health, 299 Ryrie St, Geelong, Victoria 3220, Australia; Cornell University Statistical Consulting Unit, Cornell University, Savage Hall, 244 Garden Avenue, Ithaca, New York 14853, USA; Centre for Veterinary Wildlife Research, Faculty of Veterinary Science, University of Pretoria, Private Bag X04, Onderstepoort 0110, South Africa; Department of Paraclinical Sciences, Faculty of Veterinary Science, University of Pretoria, Pathology and Veterinary Public Health Complex, Onderstepoort 0110, South Africa

**Keywords:** Butorphanol, Ceratotherium simum, etorphine, hypoxaemia, noradrenaline, sympathetic

## Abstract

White rhinoceros are a sentinel species for important ecosystems in southern Africa. Their conservation requires active management of their population, which, in turn, requires immobilization of individuals with an ultra-potent opioid such as etorphine. Unfortunately, when immobilized with etorphine, they develop severe hypoxaemia that may contribute to morbidity and mortality. We hypothesized that (i) etorphine causes sympathetic upregulation that is responsible for physiological complications that produce hypoxaemia and (ii) butorphanol, a partial μ opioid agonist, mitigates sympathetic upregulation, thereby improving arterial oxygen content (CaO_2_) and delivery (DO_2_). Six subadult male white rhinoceros were administered two treatments in random order: etorphine-saline (ES) and etorphine-butorphanol (EB). After intramuscular etorphine (~2.6 μg kg^−1^), rhinoceros became recumbent (time 0 min [t0]) and were instrumented. Baseline data were collected at t30, butorphanol (0.026 mg/kg) or 0.9% saline was administered intravenously at t37, and data were collected again at t40 and t50. At baseline, plasma noradrenaline concentration was >40 ng ml^−1^, approximately twice that of non-immobilized rhinoceros (*t* test, *P* < 0.05); cardiac output (Qt, by thermodilution) and metabolic rate (VO_2_, by spirometry/indirect calorimetry) were greater than predicted allometrically (*t* test, *P* < 0.05), and pulmonary hypertension was present. After butorphanol, noradrenaline concentration remained greater than in non-immobilized rhinoceros; in EB, CaO_2_ was greater, while Qt, DO_2_, VO_2_, and pulmonary pressures were less than in ES (linear mixed effect model, all *P* < 0.05). Increased noradrenaline concentration with increased Qt and hypermetabolism supports etorphine-induced sympathetic upregulation. Butorphanol partly attenuated these effects, increasing CaO_2_ but reducing Qt and, thus, DO_2_. Since plasma noradrenaline concentration remained increased after butorphanol administration while Qt, DO_2_, and VO_2_ decreased, a pathway independent of plasma noradrenaline concentration might contribute to the cardiopulmonary and hypermetabolic effects of etorphine. Developing treatments to combat this sympathomimesis could reduce capture-related morbidity in white rhinoceros.

## Abbreviations:

IUCN: International Union for the Conservation of Nature, IM: intramuscular, VO_2_: metabolic rate or oxygen consumption, PaO_2_: arterial partial pressure of oxygen, PaCO_2_: arterial partial pressure of carbon dioxide, Pa-aO_2_: alveolar-arterial oxygen partial pressure difference, Qt: cardiac output, SNS: sympathetic nervous system, CNS: central nervous system, IV: intravenous, f_H_: heart rate, mSAP: mean systemic arterial pressure, VCO_2_: carbon dioxide production, DO_2_: oxygen delivery to tissues, CaO_2_: arterial oxygen content, FiO_2_: fraction of inspired oxygen, ARRIVE: Animal Research: Reporting of In Vivo Experiments, KNP: Kruger National Park, ES: etorphine-saline group, EB: etorphine-butorphanol group, VWS: Veterinary Wildlife Services, LC-MS: liquid chromatography-mass spectrometry, SWG: standard wire gauge, SAP: systemic arterial blood pressure, PAC: pulmonary artery catheter, PAOP: pulmonary artery occlusion pressure, PAP: pulmonary artery pressure, PCV: packed cell volume, PēCO_2_: mixed expired carbon dioxide partial pressure, FēO_2_: mixed expired oxygen fraction, PeCO_2_: end-tidal carbon dioxide partial pressure, Vebtps: minute ventilation, at body temperature and pressure saturated, f_r_: breathing rate, Hb: haemoglobin, USA: United States of America, ODC: oxyhaemoglobin dissociation curve, Vt: tidal volume, IQR: interquartile range, SD: standard deviation, ID: identification number, PῡO_2_: mixed venous oxygen partial pressure, PVR: pulmonary vascular resistance, SV: stroke volume, OER: oxygen extraction ratio, CῡO_2_: mixed venous oxygen content, Vdphys: physiological dead space ventilation, PVN: paraventricular nucleus, CNA: central noradrenergic, LC: locus coeruleus, rVLM: rostral ventrolateral medulla, PNMT: phenylethanolamine N-methyltransferase, D_2_: dopamine 2 receptor, CRF: corticotropin releasing factor, J_v_: fluid flux, Pc: capillary hydrostatic pressure, K_f_: reflection coefficient, Πc: capillary oncotic pressure, EIPH: exercise-induced pulmonary haemorrhage, V:Q: ventilation to perfusion ratio, P_50_: oxygen partial pressure at which 50% of haemoglobin is saturated

## Introduction

The southern white rhinoceros (*Ceratotherium simum ssp. simum*) is poached heavily for its horn, and its natural range is being reduced by human encroachment. Consequently, the species is classified as ‘near-threatened’ by the International Union for the Conservation of Nature and Natural Resources (IUCN) (Emslie, 2020). To ensure survival of the species, conservationists monitor and manage white rhinoceros populations across southern Africa. Conservation strategies include translocation (to move rhinoceros to safer regions and to improve genetic heterogeneity), dehorning, collection of biological samples, fitting of tracking devices, and veterinary medical interventions, all of which require capture via chemical immobilization (Kock *et al.,* 1995; Langhout *et al.,* 2016). Thus, chemical immobilization is indispensable for conservation of the white rhinoceros.

The temperament of rhinoceros and the rugged terrain in which they live necessitates immobilization using a potent drug with a rapid onset of action, which can be delivered intramuscularly (IM) by dart from a helicopter and pharmacologically antagonized. The ultra-potent, highly lipid-soluble opioid receptor agonist, etorphine, a pure agonist at μ, δ, and κ opioid receptors, is one of only a few related drugs that fulfil these requirements and has been used to immobilize rhinoceros since the mid-20th century (Harthoorn and Bligh, 1965; Tolkovsky, 1982; Jolicoeur *et al.,* 1992; Gharagozlou *et al.,* 2006). Unfortunately, severe physiological complications, including arterial hypoxaemia, hypoventilation, respiratory and metabolic (lactic) acidaemia, tachycardia, systemic and pulmonary arterial hypertension, tremors, and increased tissue oxygen consumption (metabolic rate, VO_2_) have been documented in white rhinoceros immobilized with etorphine-based drug protocols (Haw *et al.,* 2014; Buss *et al.,* 2015; Haw *et al.,* 2015; Buss *et al.,* 2016; de Lange *et al.,* 2017; Boesch *et al.,* 2018; Buss *et al.,* 2018). These complications may cause unrecognized morbidity and result in mortality during immobilization (Kock *et al.,* 1995). Of these complications, hypoxaemia—an arterial oxygen partial pressure (PaO_2_) < 90 mm Hg—is arguably the most dangerous (Citino and Bush, 2007). Although μ opioid receptor-mediated hypoventilation, which causes hypercapnia (arterial carbon dioxide partial pressure [PaCO_2_] as high as ~80–100 mm Hg), is an important contributor to the hypoxaemia, the severity of the hypoxaemia (PaO_2_ ~ 25 mm Hg) and the increased alveolar-arterial oxygen partial pressure difference (Pa-aO_2_) observed in etorphine-immobilized white rhinoceros suggest that hypoventilation cannot solely be responsible for the hypoxaemia (Boesch *et al.,* 2018; Buss *et al.,* 2018; Saunders and Levitt, 2020). Aerial pursuit and darting in the field triggers a flight response in rhinoceros, and maximal exertion and stress during capture could explain the aforementioned physiological complications (Haw *et al.,* 2015; de Lange *et al.,* 2017). However, all of these problems have also been observed in white rhinoceros habituated to captivity in holding pens (bomas) and darted with etorphine without helicopter pursuit or adjunctive drugs (Buss *et al.,* 2016; Boesch *et al.,* 2018; Buss *et al.,* 2018). Under these conditions, exertion and stress appear to be minimal, suggesting that the etorphine alone is primarily culpable. Interestingly, under these circumstances, VO_2_ measured using indirect calorimetry is greater than predicted allometrically (Schmidt-Nielsen, 1991; Buss *et al.,* 2018).

When etorphine is administered to horses, which are phylogenetically related to rhinoceros, they develop physiological complications that resemble those observed in etorphine-immobilized white rhinoceros (Hillidge, 1971; McCue *et al.,* 2012). Hypoxaemia, hypercapnia, acidaemia, sinus tachycardia and other cardiac dysrhythmias, increased cardiac output (Qt), systemic arterial hypertension, hyperglycaemia, increased haematocrit, sweating, hypertonus of the limbs and full-body tremors are characteristic of horses, and ponies immobilized with etorphine (Hillidge, 1971; Lees and Hillidge, 1975). Increased sympathetic nervous system (SNS) activity could account for many of these observations, especially those pertaining to the cardiopulmonary system and VO_2_ (Hillidge, 1971; Daniel and Ling, 1972; Schlarmann *et al.,* 1973; Bogan *et al.,* 1978). However, the hypothesis that etorphine upregulates the SNS, causing the physiological complications observed during immobilization, has not been tested in white rhinoceros.

Although full opioid receptor antagonists such as naltrexone could be used to reverse the opioid receptor-mediated adverse effects of etorphine, they also reverse the central nervous system (CNS) effects (e.g. catatonia, sedation and analgesia) to an extent that prohibits safe handling of rhinoceros (Buss *et al.,* 2018). Therefore, in field practice, as soon as an immobilized rhinoceros is deemed safe to approach, the synthetic opioid, butorphanol, is usually administered intravenously (IV). Butorphanol is a full agonist at κ opioid receptors but a partial agonist at μ opioid receptors; at an appropriate dose, the partial agonism of the μ opioid receptor population apparently mitigates the adverse effects of etorphine, without reversing its immobilizing effects (Chang *et al.,* 1981; Garner *et al.,* 1997; Greenwald and Stitzer, 1998; Vivian *et al.,* 1999; Commiskey *et al.,* 2005). Butorphanol administration to boma-habituated white rhinoceros that have been immobilized with etorphine decreases heart rate (f_H_), mean systemic arterial pressure (mSAP), PaCO_2_, VO_2_, and carbon dioxide production (VCO_2_), and increases PaO_2_ (Buss *et al.,* 2016; Buss *et al.,* 2018).

During immobilization or anaesthesia, adequate tissue oxygenation is a priority. While many factors impact how much oxygen ultimately reaches mitochondria, oxygen delivery (DO_2_)—the product of arterial oxygen content (CaO_2_) and Qt—is an important macrocirculatory value that must be maintained within normal limits (Dodd *et al.,* 1993). If oxygen supply (i.e. DO_2_) cannot meet demand (i.e. VO_2_) by tissues, VO_2_ becomes supply-limited, oxygen debt develops, anaerobic metabolism increases and lactic (metabolic) acidaemia ensues (Nelson *et al.,* 1987; Dodd *et al.,* 1993). If oxygen debt is severe enough, cellular death occurs. Oxygen insufflation has been used to augment inspired oxygen fraction (FiO_2_) in white rhinoceros during etorphine immobilization; however, this is often impractical in the field, and it can further decrease ventilation, presumably by opposing hypoxaemia-induced ventilatory drive (Haw *et al.,* 2015). Although beneficial effects of butorphanol on PaO_2_ (and by inference, CaO_2_) have been documented, and wildlife veterinarians presently operate under the assumption that increased PaO_2_ indicates increased DO_2_ in rhinoceros, the effect of butorphanol on DO_2_ is undocumented (Buss *et al.,* 2018).

The primary objectives of our study were to determine if etorphine induces SNS upregulation in white rhinoceros and how this might lead to hypoxaemia and other physiological complications. Our secondary objective was to determine if butorphanol mitigates these pertubations and increases CaO_2_ and DO_2_. We hypothesized that in white rhinoceros (i) etorphine causes sympathetic upregulation, including increased plasma catecholamine concentrations, which is responsible for cardiopulmonary and metabolic complications that produce hypoxaemia, and (ii) the partial μ opioid agonist butorphanol mitigates the sympathetic upregulation, thereby improving CaO_2_ and DO_2_.

## Materials and Methods

### Animals

This research protocol was approved by the University of Pretoria Animal Ethics Committee (project number V101-15) and the South African National Parks (SANParks) Animal Use and Care Committee (reference number 001/16). It adheres to the Animal Research: Reporting of In Vivo Experiments (ARRIVE) guidelines (Percie du Sert *et al.,* 2020).

Six sub-adult (4–5 years old), male, white rhinoceros were used in the experiments. The immobilization and subsequent management of the rhinoceros followed the SANParks Standard Operating Procedures for the Capture, Transportation and Maintenance in Holding Facilities of Wildlife. The rhinoceros were captured via aerial darting in the Kruger National Park (KNP, 23°49′60 S, 31°30′0 E; altitude: ~317 m above sea level), South Africa. After capture, they underwent a physical examination to verify their health and were subsequently housed in groups of two or three at Veterinary Wildlife Services (VWS) bomas (dimensions: 10.5 × 21 m or 220.5 m^2^) for an habituation period of at least one month. Their diet consisted of lucerne (*Medicago sativa*) and tef (*Eragrostis tef*) hay and *ad libitum* water. Trained staff evaluated the rhinoceros daily using a scoring system developed by VWS based on the evaluation of: (i) feed intake, (ii) volume, consistency and colour of faeces, and (iii) behaviour (Miller *et al.,* 2016).

To guide the interpretation of experimental plasma catecholamine levels, venous blood samples were collected from 11 unmedicated, conscious white rhinoceros that were habituated to handling and permitted venipuncture without immobilization (i.e. 'control' white rhinoceros, Table S1). These samples were handled in the same way as the experimental samples and assayed together with them in one batch.

### Study Design

A randomized, crossover study design was used. Blinding was not possible for logistical reasons. Two treatments, etorphine followed by 0.9% (isotonic) saline (ES) and etorphine followed by butorphanol (EB), were separated by a minimum washout period of 2 weeks. *A priori*, rhinoceros were assigned to treatment order by random allocation in blocks of two by lottery, and an online randomizer (www.randomiser.org) was used to determine the order of immobilization of the rhinoceros. All experiments were conducted in the morning between 6 and 9 AM local time from September to October. Ambient temperature and pressure were measured before each immobilization (Kestrel Instruments, Boothwyn, PA, USA).

Drugs were dosed based on estimated body weight (Table S2). However, body weight was measured at the end of each experiment, and drug doses, as well as physiological variables where appropriate (below), were normalized to actual body weight for reporting and analysis. Etorphine plus hyaluronidase was delivered IM in the nuchal hump by a 3-ml plastic dart with a 60-mm uncollared needle, fired from a compressed air rifle (DAN-INJECT International S.A., Skukuza, South Africa). Hyaluronidase is used routinely to increase the rate at which etorphine is absorbed from the site of injection, and systemic effects have not been reported for hyaluronidase during capture (Kock *et al.,* 1990). As soon as a rhinoceros stood still, VWS staff experienced in the handling of white rhinoceros determined if it was safe to approach; if so, the rhinoceros was blindfolded and positioned in sternal recumbency using ropes. Sternal recumbency was designated as time 0 (t0); this was selected as t0 based on the assumption that there was parity between the rhinoceros in the level of CNS depression at this time (Buss *et al.,* 2018).

As soon as a rhinoceros was positioned in sternal recumbency, shortened, lubricated equine endotracheal tubes (KRUSSE Silicone Endotracheal Tube, internal diameter: 28 mm, no. 282270, Jørgen Kruuse A/S, Langeskov, Denmark) were inserted into the nares, and their cuffs inflated to produce an airtight seal. The rhinoceros was then positioned in left lateral recumbency, and instrumentation continued as described below. Experience indicated that instrumentation could reliably be completed within 30 min after t0, hence the first (baseline) data were collected at t30. At t37, either butorphanol (Butonil, Wildlife Pharmaceuticals Pty Ltd, White River, South Africa) or an equivalent volume of 0.9% saline was injected into an auricular vein for treatments EB and ES, respectively. Data were collected again at t40 and t50. Data collection was performed at t40, or 3 min after injection of butorphanol (or saline), to document the early effects of butorphanol. At the conclusion of the experiments, rhinoceros were de-instrumented. The rhinoceros in the EB group could be stimulated to stand and guided into a crate for weighing. The rhinoceros in the ES treatment group were given butorphanol IV so that they could be stimulated to stand and moved to the crate for weighing. After weighing, naltrexone (Kyron Laboratories Pty Ltd, Johannesburg, South Africa) was administered IV to fully antagonize the CNS effects of etorphine and butorphanol, and the rhinoceros were released back into the bomas. They were monitored continuously by trained VWS staff for the first hour after recovery and then hourly thereafter until they began eating, drinking, urinating and defaecating normally.

### Instrumentation and Data Collection

#### Plasma Catecholamine Concentrations

At each data collection time, blood was collected from an auricular vein and added to tubes containing ethylene-diamine-tetra-acetic acid anticoagulant; these were immediately put on ice and spun down in a refrigerated centrifuge at 2500 revolutions minute^−1^ for 10 min. Plasma was pipetted into cryotubes (Greiner Bio-One, Frickenhausen, Baden-Württemberg, Germany), snap-frozen in liquid nitrogen and stored at −80°C. This plasma was used to measure the concentrations of adrenaline, noradrenaline and dopamine with liquid chromatography-mass spectrometry (LC–MS, Dataset S1) (Xu *et al.,* 2018; Gao *et al.,* 2021).

#### Systemic Arterial Catheterization

After aseptic skin preparation, a 22 standard wire gauge (SWG) and 2.5-cm over-the-needle IV catheter (Nipro Medical Corporation, Bridgewater, NJ, USA) were inserted into a medial auricular artery for measurement of SAP at each data collection time. The catheter was connected by non-compliant tubing to a pressure transducer (Deltran II pressure transducer, DPT-200, Utah Medical Products Inc., Midvale, UT, USA) zeroed to atmospheric pressure at the level of the *manubrium sterni*; the entire system was filled with 0.9% saline. The transducer was connected to an amplifier (BP Amp, FE117, ADInstruments Pty Ltd., New South Wales, Australia) and a digital acquisition system (PowerLab 8/30, ADInstruments Pty Ltd).

#### Pulmonary Arterial Catheterization

A catheter introducer was placed aseptically into a linguofacial vein using a technique that was described previously (Boesch *et al.,* 2018). A custom-built, 200 cm, 7 Fr (2.33 mm), steel braid-reinforced, polyurethane Swan-Ganz-type (balloon-tipped) thermodilution pulmonary arterial catheter (PAC, Gaeltec Devices, Dunvegan, Isle of Skye, Scotland) was then inserted through the introducer into the jugular vein and passed into the right atrium, right ventricle and pulmonary artery (Swan *et al.,* 1970). The PAC contained two lumens, each with a diameter of 0.75 mm. The proximal lumen was used to inflate a balloon located 3 cm from the PAC tip; the volume of air injected was sufficient to inflate the balloon to a diameter of 2–3 cm at ambient pressure. The distal lumen, which opened at the PAC tip, was connected to an arrangement similar to that used for measuring and recording SAP (above). Real-time observation of the blood pressure waveform allowed confirmation of the location of the tip of the PAC as it was introduced (Milne *et al.,* 1975). Once a pulmonary arterial pressure (PAP) waveform was observed, the PAC was advanced until the inflated balloon wedged in a branch of the pulmonary artery and a pulmonary arterial occlusion pressure (PAOP) waveform was observed on the monitor. Mean PAP (mPAP) and mean PAOP (mPAOP, recorded within 10 s of wedging) were measured at each data collection time. The balloon was then deflated, and the PAC was withdrawn slightly and left with the tip in the pulmonary artery so that it was ready for subsequent pressure measurements. The thermistor of the PAC was located just proximal to the balloon.

#### Cardiac Output

The PAC thermistor was connected to a cardiac output monitor (PM-9000 Vet Veterinary Portable Multi-Parameter Patient Monitor, ShenZhen Mindray Bio-Medical Electronics Co., Nanshan, ShenZhen, China). To calculate Qt, the software in this device applied Stewart–Hamilton principles to the temperature–time curve generated by a bolus of ice-cold 0.9% saline that had been injected into the linguofacial vein introducer and transited the pulmonary artery. The software required manual input of a thermistor-specific computation constant. By design, the thermistor in the PAC had properties identical to those used in a commercial thermodilution catheter (ARROW Balloon Thermodilution catheter, Teleflex, Morrisville, NC, USA), allowing use of the computation constant for those commercial catheters. The thermistor was also used to measure pulmonary artery temperature (in °C) prior to measurement of Qt. For each Qt measurement, 60 ml of ice-cold 0.9% saline was hand injected by the same investigator at end-expiration. Three to five sequential measurements within 20% of each other were recorded at each data collection time, and the median value was recorded for analysis.

#### Blood Gas and Acid-Base Analysis and Packed Cell Volume

Arterial and mixed venous blood samples were collected simultaneously from the systemic arterial catheter and the distal port of the PAC, respectively, at each data collection time. Blood samples (1 ml each) were collected anaerobically into 2-ml heparinized syringes, put on ice, and analyzed immediately with a point-of-care analyser (epoc Blood Analysis System, Siemens Medical Solutions, Inc., Malvern, PA, USA). Values for pH and blood gas partial pressures at 37°C were recorded. A portion of each sample was centrifuged at 15,000 g for 10 minute to obtain packed cell volume (PCV).

#### Spirometry and Indirect Calorimetry

The nasal tubes described above were connected to a valve system that allowed inspiration of ambient air and directed all expired gas into a mixing chamber (MLA245, ADInstruments Pty Ltd.), where its temperature was measured (Thermistor Temperature Sensor, MLT415/M, ADInstruments Pty Ltd.), and then through a pneumotachometer (Respiratory Flow Head 1000L, MLT300L, ADInstruments Pty Ltd.) (Buss *et al.,* 2018). Expired gas was released into the atmosphere except for a period of 1 minute at the beginning of each of the three data collection times, when a Douglas bag was attached to the pneumotachometer for collection of the mixed expired gas (Buss *et al.,* 2018). Mixed expired carbon dioxide partial pressure (PēCO_2_, mm Hg) and mixed expired oxygen fraction (FēO_2_, %) were measured in the Douglas bag (Cardiocap/5 monitor, Datex-Ohmeda, GE Healthcare, Helsinki, Finland). This device was also used to measure end-tidal carbon dioxide partial pressure (PeCO_2_) from a sampling port in one of the nasal tubes just outside the naris.

#### Tremor Score

Skeletal muscle activity was scored at each data collection time by a single experienced, unblinded observer using a previously published tremor scoring system (Table S3) (Buss *et al.,* 2018).

#### Spirometry and Blood Pressure Waveform Acquisition and Analysis

Spirometry and blood pressure waveforms were processed using dedicated hardware (PowerLab 8/30, ML870, ADInstruments Pty Ltd., New South Wales, Australia) and acquired digitally for subsequent analysis (LabChart 7 data acquisition software with Metabolic Module, version 7.3.8, ADInstruments Pty Ltd) on a laptop computer. Expired minute ventilation, corrected to pulmonary artery temperature and pressure, saturated with water vapor (Vebtps) and respiratory rate (f_R_) were measured from the expiratory flow waveform displayed by LabChart 7 (Boesch, 2020). The mPAP, mPAOP, mSAP and f_H_ were measured from the pulmonary and systemic arterial pressure waveforms also displayed by LabChart 7 (Boesch, 2020).

### Calculations

Since a method for measuring haemoglobin concentration ([Hb]) was unavailable, we generated an equation for calculating [Hb] from PCV in the white rhinoceros. Between 2013 and 2017, 214 sub-adult, male white rhinoceros were captured in KNP. On a sample of blood from each animal, the PCV was measured as described above, and the cyanmethaemoglobin method was used to measure the corresponding [Hb] in an automated analyser (Vet abc, scil Animal Care Company, Gurnee, IL, USA) (Prakash and Banerji, 1972; Whitehead *et al.,* 2019). A regression line (described by the equation *y* = *mx* + *b*, with *m* = slope and *b* = *Y* intercept) for PCV vs. [Hb] was generated using a commercial software package (GraphPad Prism 7, GraphPad Software Inc., San Diego, CA, USA). This line was described by the equation *y* = 0.2528*x* + 4.013; the value for *r*^2^ was 0.535. This equation was used to calculate [Hb] from the measured PCV of the arterial and mixed venous samples.

Arterial and mixed venous oxyhaemoglobin saturations were calculated using an equation for the white rhinoceros-specific oxyhaemoglobin dissociation curve (ODC) (Reiners *et al.,* 2019). The equations for calculating other physiological variables are listed in Table S4. Allometric estimates for normal VO_2_, Qt, f_H_, Vebtps, f_R_, tidal volume (Vt) and were calculated for each animal (Schmidt-Nielsen, 1991).

### Statistics

#### Sample Size Calculation

Sample size was calculated *a priori* with a two-tailed *t* test of the difference in PaO_2_ values between treatments in a similar crossover study in white rhinoceros (Buss *et al.,* 2018). Median PaO_2_ in each treatment in the latter study was used in place of mean PaO_2_, and interquartile range (IQR) in each treatment was used to calculate standard deviation (SD) (Wan *et al.,* 2014). Median and IQR values were entered into commercial software (G*Power 3.1.9.2, Kiel University, Kiel, Germany), with correlation between treatments set at 0.5. This produced an effect size estimate (Cohen’s *d_z_*) of 3.408. With α = 0.05 and power (1–ß) of 0.8, a sample size of three was needed. We used six rhinoceros in anticipation of loss of data for technical reasons.

#### Data Analysis

Data analysis was performed using commercial software (JMP 14.0.0, SAS Institute, Inc., Cary, NC, USA). No data were excluded from analysis. After performing a Shapiro-Wilk test to confirm normality of the data, a paired *t* test was used to compare ambient temperature and pressure, body weight of the rhinoceros, etorphine dose and time to sternal recumbency in the two treatments; alpha was set at 0.05. Residuals were calculated, and the Shapiro–Wilk test was used to confirm that the residuals were normally distributed.

For each of the physiological variables measured or calculated at t30, t40 or t50, a linear mixed effect model was constructed, with each variable designated as the response (‘dependent’) variable. Time, treatment (or ‘phase’), and the interaction of time and treatment (‘phase’) were designated as fixed effects, and rhinoceros identification number (ID) and rhinoceros ID nested within treatment were designated as random effects. *A priori*, catecholamine concentrations, mPAP, mPAOP, Qt, PaO_2_, mixed venous oxygen partial pressure (PῡO_2_), DO_2_ and VO_2_, were designated as the ten primary outcomes. The remaining variables were designated as secondary outcomes. Alpha was set at 0.05 for the primary outcomes, and 0.05/19 = 0.0025 for the 19 secondary outcomes (a Bonferroni correction for multiple comparisons). For each variable, the 36 residuals (three data collection times × two treatments × six rhinoceros) were calculated, and the Shapiro–Wilk test was used to confirm that the residuals were normally distributed. Residuals for pulmonary vascular resistance (PVR), stroke volume (SV), Pa-aO_2_ and f_R_ were not normally distributed; thus, the data for these variables were log-transformed, and residuals were re-tested to confirm normality. Residuals for log-transformed Pa-aO_2_ data were not normally distributed; however, the fit model was retained for this single variable.

A paired *t* test (two-tailed) was used to measure the significance of differences between measured VO_2_, Qt, f_H_, f_R_, Vebtps and Vt at t30 for treatment ES and the corresponding values calculated allometrically. An unpaired *t* test (two-tailed) was used to measure the significance of differences in the plasma catecholamine concentrations between the ES experimental rhinoceros at t30 and the non-immobilized white rhinoceros. Alpha was set at 0.05. Data are expressed as mean ± SD.

## Results

During treatments ES and EB, respectively, ambient temperature was 22.4 ± 2.2 and 23.0 ± 3.8°C, atmospheric pressure was 740.7 ± 4.1 and 742.1 ± 2.3 mm Hg, and body weight of the rhinoceros was 1145 ± 73 and 1152 ± 98 kg. Etorphine dose administered was 2.6 ± 0.2 and 2.6 ± 0.1 μg kg^−1^. Time to sternal recumbency was 12.4 ± 2.0 and 11.7 ± 2.0 min. There were no significant differences between treatments for any of the above variables. No complications arose from PAC insertion, and the catheter was long enough to permit wedging in all rhinoceros at all data collection times. All rhinoceros recovered from the procedures and were subsequently released into the wild.

**Figure 1:**
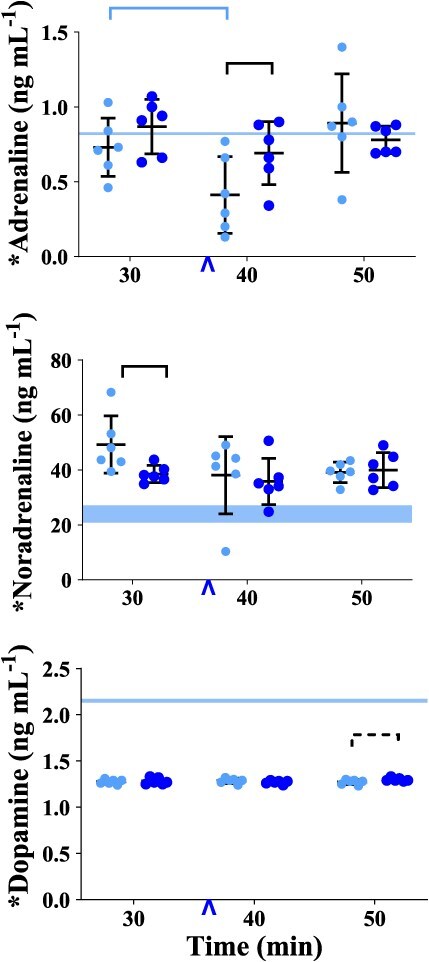
Graphs of plasma catecholamine concentrations measured in healthy, boma-habituated, sub-adult, male white rhinoceros (*n* = 6), assigned to two treatments administered once each in random order: etorphine-saline (ES, light blue circles) and etorphine-butorphanol (EB, dark blue circles). Blood was collected 30, 40 and 50 min after the rhinoceros became recumbent (t30, t40 and t50, respectively); either 0.9% saline (treatment ES) or butorphanol (treatment EB) were injected IV at t37. The central line within each data set represents the mean, whereas the error bars represent the SD. Refer to Figs 2–6 for graphs of other variables measured or calculated in the rhinoceros. A linear mixed effect model was constructed to assess changes in each of the 29 variables over time within treatments and between treatments. For the purposes of statistical analysis, the ten variables labelled with * on the *Y*-axis were considered primary outcomes; the 19 others were considered secondary outcomes. For the primary outcomes, alpha was set at 0.05; for the secondary outcomes, alpha was set at 0.05/19 = 0.0026 (a Bonferroni correction for multiple comparisons). In all figures, a solid bar connecting data sets indicates a significant difference, and a dashed bar connecting data sets indicates a significant difference before Bonferroni correction. NB: Differences that lost significance after Bonferroni correction were not considered significant for the purposes of discussion in this paper. The blue arrowhead on the *X*-axis indicates when saline or butorphanol was administered at t37. The light blue band on the *Y*-axis of certain graphs indicates the 95% confidence interval for the catecholamine values measured in the non-immobilized rhinoceros or the allometrically calculated values for the etorphine-immobilized rhinoceros.

**Figure 2:**
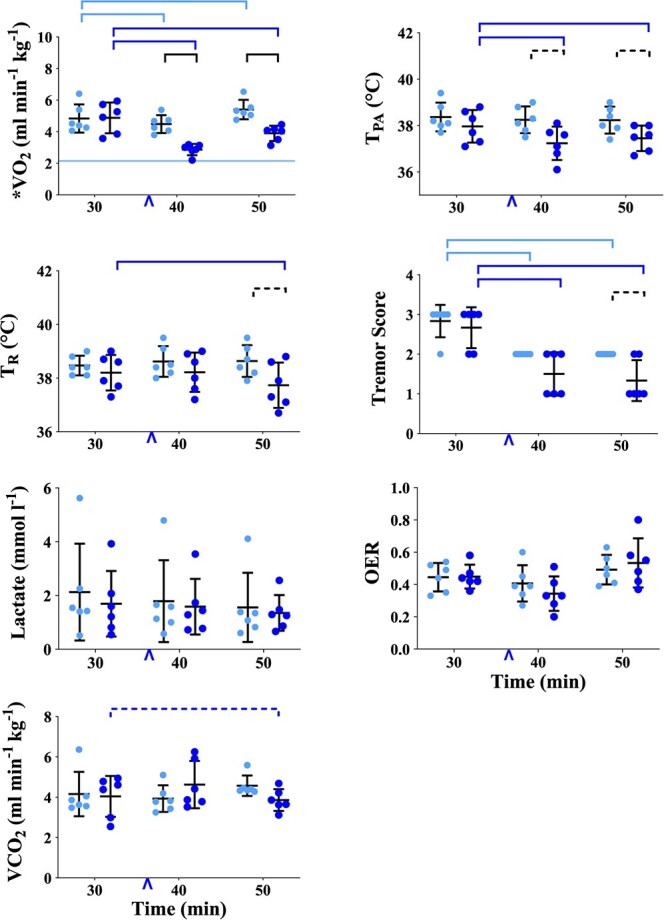
Graphs of physiological variables measured or calculated in healthy, boma-habituated, sub-adult, male white rhinoceros (*n* = 6), assigned to two treatments administered once each in random order: ES (light blue circles) and EB (dark blue circles). Data were collected 30, 40 and 50 min after the rhinoceros became recumbent (t30, t40 and t50, respectively); either 0.9% saline (treatment ES) or butorphanol (treatment EB) was injected IV at t37. Refer to Fig. 1 legend for further details. VO_2_, oxygen consumption; T_PA_, pulmonary artery temperature; T_R_, rectal temperature; OER, oxygen extraction ratio; VCO_2_, carbon dioxide production.

**Figure 3:**
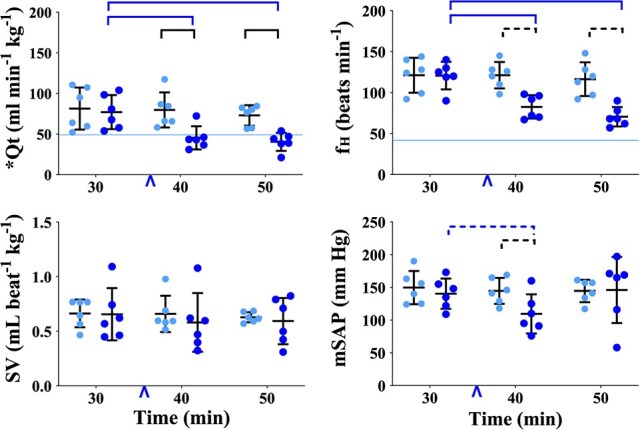
Graphs of physiological variables measured or calculated in healthy, boma-habituated, sub-adult, male white rhinoceros (*n* = 6), assigned to two treatments administered once each in random order: ES (light blue circles) and EB (dark blue circles). Data were collected 30, 40 and 50 min after the rhinoceros became recumbent (t30, t40 and t50, respectively); either 0.9% saline (treatment ES) or butorphanol (treatment EB) was injected IV at t37. Refer to Fig. 1 legend for further details. Qt, cardiac output; f_H_, heart rate; SV, stroke volume; mSAP, mean systemic arterial pressure.

**Figure 4:**
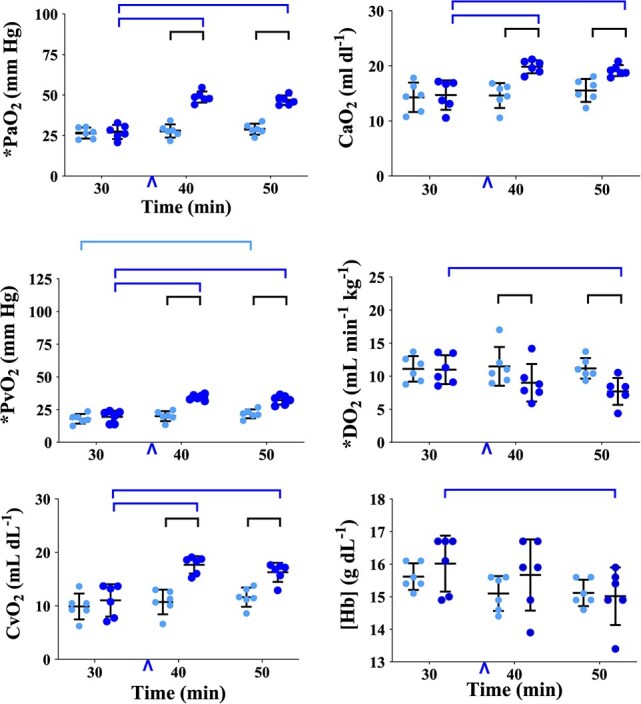
Graphs of physiological variables measured or calculated in healthy, boma-habituated, sub-adult, male white rhinoceros (*n* = 6), assigned to two treatments administered once each in random order: ES (light blue circles) and EB (dark blue circles). Data were collected 30, 40 and 50 min (min) after the rhinoceros became recumbent (t30, t40 and t50, respectively); either 0.9% saline (treatment ES) or butorphanol (treatment EB) were injected IV at t37. Refer to Fig. 1 legend for further details. PaO_2_, arterial oxygen partial pressure; CaO_2_, arterial oxygen content; PῡO_2_, mixed venous oxygen partial pressure; DO_2_, oxygen delivery; CῡO_2_, mixed venous oxygen content; [Hb], haemoglobin concentration.

**Figure 5:**
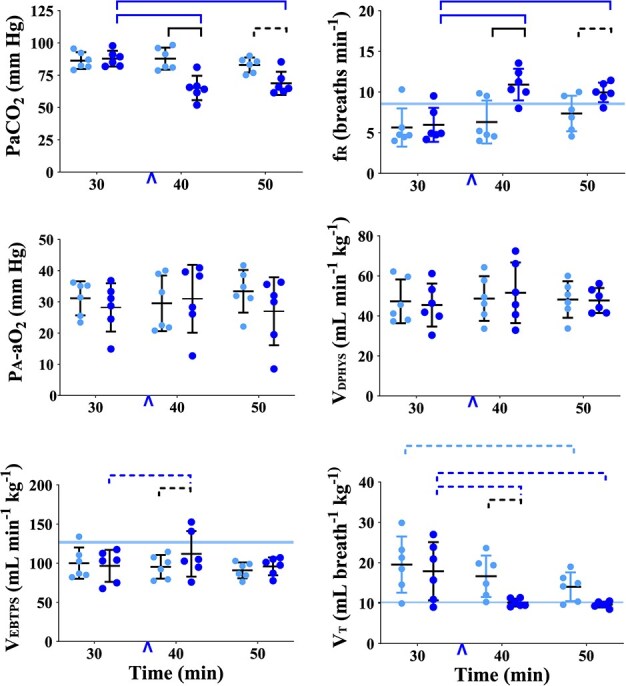
Graphs of physiological variables measured or calculated in healthy, boma-habituated, sub-adult, male white rhinoceros (*n* = 6), assigned to two treatments administered once each in random order: ES (light blue circles) and EB (dark blue circles). Data were collected 30, 40 and 50 min after the rhinoceros became recumbent (t30, t40 and t50, respectively); either 0.9% saline (treatment ES) or butorphanol (treatment EB) was injected IV at t37. Refer to Fig. 1 legend for further details. PaCO_2_, arterial carbon dioxide partial pressure; f_R_, respiratory rate; Pa-aO_2_, alveolar-arterial oxygen partial pressure difference; Vdphys, physiological dead space ventilation; Vebtps, minute ventilation, body temperature and pressure saturated with water vapour; Vt, tidal volume.

**Figure 6:**
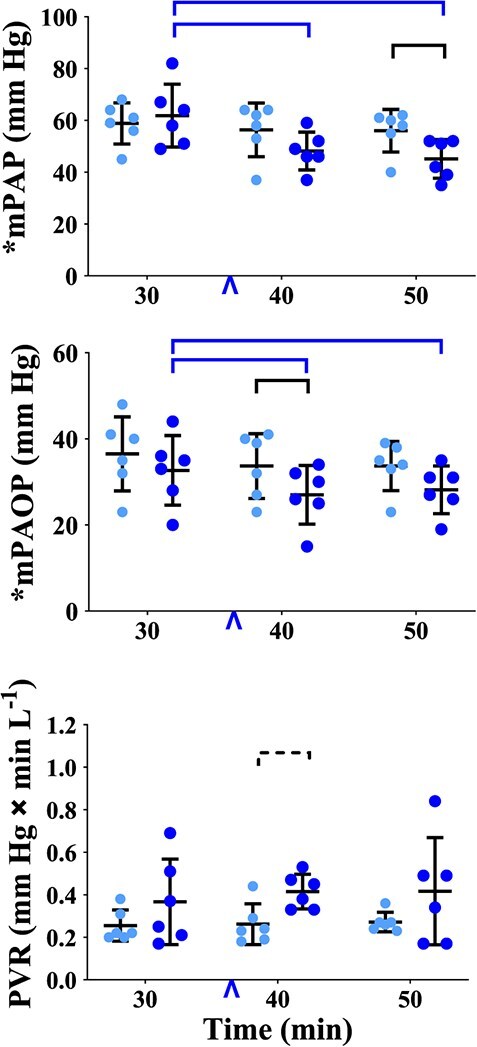
Graphs of physiological variables measured or calculated in healthy, boma-habituated, sub-adult, male white rhinoceros (*n* = 6), assigned to two treatments administered once each in random order: ES (light blue circles) and EB (dark blue circles). Data were collected 30, 40 and 50 min after the rhinoceros became recumbent (t30, t40 and t50, respectively); either 0.9% saline (treatment ES) or butorphanol (treatment EB) were injected IV at t37. Refer to Fig. 1 legend for further details.

Changes in physiological variables over time within each treatment and between treatments are shown in Figs 1-6 and Table S5. At t30, before saline or butorphanol was administered, no significant differences were found between treatments ES and EB for any variable except noradrenaline concentration, which was significantly greater in the former at t30.

In the group of non-immobilized white rhinoceros, the plasma concentrations of adrenaline, noradrenaline and dopamine were 0.82 ± 0.01, 23.92 ± 4.67 and 2.15 ± 0.03 ng ml^−1^, respectively (Table S2). In these rhinoceros, one plasma noradrenaline concentration (11.28 ng ml^−1^) was identified as an outlier; it was not excluded from the analysis since we had no explanation to support doing so.

### Effects of Etorphine (Treatment ES)

#### Plasma Catecholamine Concentrations

Compared to the non-immobilized rhinoceros, plasma noradrenaline concentrations were greater, plasma dopamine concentrations were less, and plasma adrenaline concentrations did not differ significantly. At t30 in treatment ES, the mean plasma noradrenaline concentration (49.25 ± 10.44 ng ml^−1^) was approximately twice that observed in non-immobilized rhinoceroses (*P* = 0.0012). The mean plasma dopamine concentration (1.28 ± 0.03 ng ml^−1^) was lower than in the non-immobilized group (*P* < 0.0001), while plasma adrenaline concentrations (0.73 ± 0.19 ng ml^−1^) did not differ significantly from those of non-immobilized rhinoceros (*P* = 0.3050).

Plasma adrenaline concentration was transiently less at t40, but plasma noradrenaline and dopamine concentrations did not change between t30 and t50 (Fig. 1, Table S5). Within treatment ES, plasma adrenaline concentration decreased between t30 and t40 (*P* = 0.0369), but then rebounded between t40 and t50. Plasma noradrenaline and dopamine concentrations did not change significantly over time.

#### Allometric Comparisons

At t30, metabolic rate (oxygen consumption) and cardiac output were greater and ventilation was less than predicted allometrically. At t30, in treatment ES, VO_2_, Qt, f_H_ and Vt were 129, 65, 195 and 92% greater, respectively, than predicted allometrically; f_R_ and Vebtps were 34 and 21% less, respectively, than predicted allometrically (Table 1).

**Table 1:** Values [mean ± SD] for physiological variables measured in boma-habituated, subadult, male white rhinoceros (*n* = 6) immobilized with etorphine (treatment ES) 30 min after sternal recumbency (t30) and corresponding normal values (mean ± SD) calculated allometrically from body weight

	VO_2_ (ml min^−1^ kg^−1^)	Qt (ml min^−1^ kg^−1^)	f_H_ (beats min^−1^)	f_R_ (breaths min^−1^)	Vebtps (ml kg^−1^)	V_T_ (ml kg^−1^)
Mean ± SD (measured at t30 in treatment ES)	4.8 ± 0.89	81 ± 26	121 ± 21	5.6 ± 2.3	100 ± 20	19.6 ± 7.0
Mean ± SD (calculated allometrically)	2.1 ± 0.03	49 ± 0.61	42 ± 0.82	8.6 ± 0.16	127 ± 2	10.2 ± 0.0
*P* value	0.0007^a^	0.027^a^	0.0003^a^	0.04^a^	0.02^a^	0.026^a^

^a^Significant difference (*P* < 0.05) between measured and allometrically calculated values. VO_2_, oxygen consumption, standard temperature and pressure dry (STPD); Qt, cardiac output; f_H_, heart rate; f_R_, breathing rate; Vebtps, expired minute ventilation, body temperature and pressure saturated with water vapour (BTPS); Vt, tidal volume.

#### Physiological Variables Over Time

At t40 and t50, values for most physiological variables were not different from values at t30. The exceptions were VO_2_ (Fig. 2, Table S5), which was less at t40 (*P* = 0.0136) and greater at t50 (*P* = 0.0082); tremor score (Fig. 2, Table S5), which was less at both t40 and t50 (*P* < 0.0001 for both); and PῡO_2_ (Fig. 4, Table S5), which was higher at t50 (*P* = 0.0018).

### Effects of Butorphanol (Treatment EB) Over Time and Compared With Treatment ES

#### Catecholamine concentrations

*Within treatment EB, the plasma catecholamine concentrations did not change significantly over time* (Fig. 1, Table S5). At t40, plasma adrenaline concentration was greater in treatment EB than ES (*P* = 0.0366). At t30, before butorphanol was administered, plasma noradrenaline concentration was less in treatment EB than ES (*P* = 0.0338).

#### Metabolic variables

*Butorphanol attenuated oxygen consumption and tremors.* Within treatment EB, VO_2_ (Fig. 2, Table S5) was less at both t40 and t50 than at t30 (*P* < 0.0001 for both). At t40 and t50, VO_2_ was less in treatment EB than ES (*P* = 0.0009 and 0.0029, respectively). Within treatment EB, tremor score (Fig. 2, Table S5) was less at both t40 and t50 than at t30 (*P* < 0.0001 for both). Butorphanol reduced both pulmonary artery and rectal temperatures. Within treatment EB, pulmonary artery temperature (Fig. 2, Table S5) was less at both t40 and t50 than at t30 (*P* < 0.0001 for both), whereas rectal temperature (Table S5) was less at t50 than at t30 (*P* < 0.0001).

Plasma lactate concentration, oxygen extraction ratio (OER), and VCO_2_ (Fig. 2, Table S5) did not change over time and were not different between treatments.

#### Cardiovascular Variables

*Butorphanol reduced cardiac output and heart rate*. Within treatment EB, Qt (Fig. 3, Table S5) was less at both t40 and t50 than at t30 (*P* = 0.0004 and 0.0001, respectively). At t40 and t50, Qt was less in treatment EB than in ES (*P* < 0.0001 for both). Within treatment EB, f_H_ (Fig. 3, Table S5) was less at both t40 and t50 than at t30 (*P* < 0.0001 for both).

*Butorphanol increased arterial and mixed venous oxygen partial pressure and content*. Within treatment EB, PaO_2_, PῡO_2_, CaO_2_ and mixed venous oxygen partial pressure (CῡO_2_) (Fig. 4, Table S5) were greater at both t40 and t50 than at t30 (all *P* < 0.0001). At t40 and t50, all were greater in treatment EB than ES (all *P* < 0.0001).

*Butorphanol reduced oxygen delivery to tissues*. Within treatment EB, DO_2_ (Fig. 4, Table S5) was less at t50 than at t30 (*P* = 0.0091). At both t40 and t50, DO_2_ was less in treatment EB than ES (*P* = 0.0173 and 0.0015, respectively). Blood haemoglobin concentration [Hb] (Fig. 4, Table S5) was less at t50 than at t30 (*P* = 0.0003).

Values for SV and mSAP (Fig. 3, Table S5) did not change over time and were not different between treatments.

#### Pulmonary variables

*Butorphanol increased breathing rate and reduced PaCO_2_*. Within treatment EB, f_r_ (Fig. 5, Table S5) was greater at both t40 and t50 than at t30 (*P* = 0.0001 and 0.0003, respectively). At t40, f_R_ was greater in treatment EB than ES (*P* = 0.0012). Within treatment EB, PaCO_2_ (Fig. 5, Table S5) was less at both t40 and t50 than at t30 (*P* < 0.0001 for both). At t40, PaCO_2_ was less in treatment EB than ES (*P* = 0.0005) (Fig. 5, Table S5).

*Butorphanol reduced pressure in the pulmonary vascular system.* Within treatment EB, mPAP (Fig. 6, Table S5) was less at both t40 and t50 than at t30 (*P* = 0.0007 and 0.0001, respectively). At t50, mPAP was less in treatment EB than ES (*P* = 0.0153). Within treatment EB, mPAOP (Fig. 6, Table S5) was less at both t40 and t50 than at t30 (*P* = 0.0031 and 0.0145, respectively). At t40, mPAOP was less in treatment EB than ES (*P* = 0.0458).

The Pa-aO_2_, physiological dead space ventilation (Vdphys), Vebtps, Vt (all Fig. 5, Table S5) and PVR (Fig. 6, Table S5) did not change over time and were not different between treatments.

## Discussion

Previous studies suggested that assessing sympathetic activity and calculating DO_2_ would help elucidate the effects of the ultra-potent opioid agonist etorphine with a view to identifying ways to minimize its adverse effects in white rhinoceros. This study measured plasma catecholamines and used modifications of established techniques to measure Qt, pulmonary arterial pressures, core (pulmonary artery) temperature and PῡO_2_ for the first time in this species. Application of the species-specific ODC allowed other variables to be derived for the first time, including CaO_2_, CῡO_2_, DO_2_ and OER (Reiners *et al.,* 2019). Butorphanol, a partial μ opioid agonist and κ opioid agonist, is used to mitigate the side effects of etorphine in white rhinoceros. Thus, this study aimed to investigate the mechanisms by which butorphanol might clarify the side effects of etorphine.

### Effects of Etorphine in White Rhinoceros

As observed previously, etorphine-induced hypoxaemia, hypercapnia, tachycardia, systemic arterial hypertension, tremors, increased VO_2_ and hyperthermia (Buss *et al.,* 2018).

#### Plasma Catecholamine Concentrations

Under etorphine, plasma noradrenaline concentration was greater than that measured in non-immobilized rhinoceros, and Qt, f_H_ and VO_2_ were substantially greater than predicted allometrically (Schmidt-Nielsen, 1991). These observations are consistentwith etorphine-induced SNS upregulation in white rhinoceros. Noradrenaline is the neurotransmitter of the SNS, but when a threshold plasma concentration is exceeded (1.8 ng ml^−1^ in humans), it also acts as a hormone with important cardiopulmonary and metabolic effects that are exhibited during the fight-or-flight and stress responses, the extent of which are related to its plasma concentration (Silverberg *et al.,* 1978). Thus, it is likely that the high plasma noradrenaline concentrations measured in this study contributed to the cardiopulmonary and metabolic side effects observed in etorphine-immobilized white rhinoceros.

The mechanism responsible for the etorphine-induced plasma noradrenaline concentrations is unclear. While hypoxaemia and hypercapnia can elevate noradrenaline in other mammals, their effect is minor compared to the levels seen in these rhinoceros (Rose *et al.,* 1983). Evidence from normoxic and normocapnic rats shows that opioids can induce catecholamine release and clinical signs of SNS activation, such as tachycardia and hypertension, suggesting that mechanisms other than hypoxaemia and hypercapnia may contribute to this response (Feuerstein and Siren, 1988).

Opioid-induced mechanisms, central and peripheral, that might explain sympathetic upregulation have been described in other species (Rose *et al.,* 1983; Chen *et al.,* 1999). In rats, β endorphins—endogenous opioid peptides that act as agonists at opioid receptors – increase central sympathetic outflow to the adrenal medulla and peripheral sympathetic nerve endings, thus stimulating peripheral catecholamine release and increasing plasma catecholamine concentrations (van Loon *et al.,* 1981). In humans, IV injection of both μ (fentanyl and morphine) and κ (nalbuphine) opioid receptor agonists induce significant, dose-dependent increases in plasma noradrenaline and adrenaline concentrations in healthy male individuals (Hoehe and Duka, 1993). Opioid receptors have been identified in the hypothalamus, the primary brain centre governing neuroendocrine and autonomic functions (Quirion *et al.,* 1983). Sympathetic outflow is increased by injection of low doses of μ opioid receptor agonists into nuclei of the hypothalamus, including the paraventricular nuclei (PVN), of unanaesthetized rats (Feuerstein *et al.,* 1983; Hassen *et al.,* 1984; Kiritsy-Roy *et al.,* 1986; Siren *et al.,* 1989; Siren and Feuerstein, 1991; Bachelard *et al.,* 1997). The hypothalamic PVN project directly to central noradrenergic (CNA) sites in the brainstem, including the locus ceruleus (LC) in the pons and the rostral ventrolateral medulla (rVLM) (Koba *et al.,* 2018). Etorphine’s lipid solubility would allow it to cross the blood-brain barrier and reach these sites rapidly (Jolicoeur *et al.,* 1992). In turn, these CNA sites project to the cell bodies of the preganglionic SNS neurons in the intermediolateral cell column of the thoracolumbar spinal cord; after synapsing with post-ganglionic SNS neurons in various peripheral ganglia, post-ganglionic neurons terminate in organs throughout the body, notably the heart and adrenal medulla (Ross *et al.,* 1984; Strack *et al.,* 1989; Ally, 1998; Koba *et al.,* 2018).

The adrenal medulla is the principal source of plasma catecholamines (Kopin, 1968). All three opioid receptors (μ, κ and δ) have been identified in the adrenal medulla, with the concentration of each dependent on species (Dumont and Lemaire, 1984; Mansour *et al.,* 1986). Catecholamine synthesis occurs in the chromaffin cells of the medulla, beginning with the production of dopamine from dopa (Nagatsu *et al.,* 1964; Cahill *et al.,* 1996). Noradrenaline is then synthesized from dopamine by dopamine-β-hydroxylase (Levin *et al.,* 1960). Finally, adrenaline is synthesized from noradrenaline by phenylethanolamine N-methyltransferase (PNMT) (Cahill *et al.,* 1996). Catecholamines are stored in the chromaffin cells and secreted when tone increases in the SNS (Coupland, 1965; Cahill *et al.,* 1996). In the etorphine-immobilized rhinoceros at t30, compared to concentrations in non-immobilized rhinoceros, adrenaline concentration was similar, dopamine concentration was less, and noradrenaline concentration was greater. Differential secretion of catecholamines depending on which opioid is administered has been documented in people; in healthy male humans, fentanyl and nalbuphine increased plasma adrenaline and noradrenaline concentrations, while morphine increased noradrenaline only (Hoehe and Duka, 1993). This is consistent with our observations in white rhinoceros that adrenaline concentrations were unremarkable under etorphine, while noradrenaline concentrations were apparently increased. The explanation for differential secretion under μ opioid agonists may lie in the two populations of chromaffin cells that have been identified in the adrenal medulla: one in which 81% of the catecholamine content is adrenaline, and another where 75% of the catecholamine content is noradrenaline (Cahill *et al.,* 1996). The population of cells with the greater adrenaline content also has a greater concentration of the enzyme PNMT (Cahill *et al.,* 1996). This enzyme is induced by glucocorticoids; under chronic stress, glucocorticoids are transported from the adrenal cortex to the medulla in uniquely high concentrations by an intra-adrenal portal system, thus upregulating PNMT, increasing adrenaline synthesis, and thereby making more of it available for secretion (Wurtman, 2002). When stressors, and particularly chronic stress, are minimal, as we assume was the case with these boma-habituated white rhinoceros, it is possible that PNMT was not unduly induced, thus prioritizing noradrenaline secretion over adrenaline secretion during etorphine-induced sympathomimesis. It is interesting that white rhinoceros that were darted with etorphine in the field while being pursued by a helicopter had plasma concentrations of noradrenaline that are less than reported here and adrenaline concentrations that were higher (de Lange *et al.,* 2017). The explanation for this difference might lie in the greater stress, which those rhinoceros were experiencing while being chased but could also be due to the tranquilizer azaperone, a dopamine receptor (D_2_) antagonist and α_1_ adrenoceptor antagonist, which was co-administered with etorphine to those rhinoceros (de Lange *et al.,* 2017).

Although these boma-habituated rhinoceros did not appear to be under stress, it is worth noting that all these samples were collected after at least one previous immobilization/capture with etorphine; half of the data was collected several weeks after a single injection of etorphine during capture in the wild, and the other half of the data was collected after a second etorphine injection during the second phase of the crossover study. The prior darting episodes might constitute an escalating allostatic load (defined as the cumulative effect of stress on the body caused by several attempts to adapt to stressors over time) that could have sensitized the rhinoceros, exaggerating normal bio-behavioural responses and generated a self-sustaining feed-forward loop involving outpouring of corticotropin-releasing hormone (CRF), a product of the hypothalamus, and noradrenaline (Elman and Borsook, 2019). However, inspection of plasma noradrenaline concentration in the rhinoceros after the first and second immobilizations showed that it was actually greater in five of six rhinoceros after the first rather than the second immobilization at t30. This seems not to support the theory of increasing allostatic load.

#### Metabolic Effects

##### Muscle Tremors

The tremors observed in etorphine-immobilized white rhinoceros might have been triggered by noradrenaline agonism of β_2_ receptors in skeletal muscle (Marsden and Meadows, 1970; Kania, 1977). However, central mechanisms cannot be excluded, such as the antidopaminergic effect of etorphine in the CNS, which produces tremors in rats (Kania, 1977). In support of the latter mechanism, it should be noted that the rhinoceros under etorphine had lower plasma dopamine concentrations than the non-immobilized, ‘control’ cohort. It should also be noted that tremors decreased over time in the ES group while a corresponding decrease in plasma nordarenaline was not detected; this lends further support for a central mechanism, perhaps related to etorphine metabolism and decreasing CNS concentration. In any case, the skeletal muscle activity from the tremors would have contributed to the greater than predicted VO_2_ and hyperthermia under etorphine.

##### Oxygen Extraction Ratio

The oxygen extraction ratio (OER) (or VO_2_ ÷ DO_2_) in these etorphine-immobilized rhinoceros was approximately twice the normal range of 20–30% reported in resting foals and horses, likely contributing to the observed low CῡO_2_ (Cambier *et al.,* 2008; Wong *et al.,* 2017). Greater than normal OER results from increasing VO_2_ and/or decreasing DO_2_. In these rhinoceros immobilized with etorphine, as has been established previously, VO_2_ is two times greater than expected (Schmidt-Nielsen, 1991; Buss *et al.,* 2018). Decreased DO_2_, the product of CaO_2_ and Qt, can result from decreased CaO_2_ and/or decreased Qt. In these etorphine-immobilized rhinoceros, CaO_2_ was substantially less than normal in other mammals, including domestic horses, while Qt was substantially greater than predicted allometrically; the increased Qt tends to cancel the decreased CaO_2_ (Schmidt-Nielsen, 1991; Butler *et al.,* 1993). Because DO_2_ has not been measured in rhinoceros that are not immobilized, and allometric values are unavailable for this variable in this species, our data do not allow us to confidently say if DO_2_ is in fact decreased abnormally. Lactic acidemia ordinarily ensues if oxygen supply (i.e. DO_2_) cannot meet oxygen demand (i.e. VO_2_). Although the mean plasma lactate concentration in etorphine-immobilized rhinoceros was elevated compared to resting horses (2.0 vs. 0.5 mmol l^−1^), it was lower than concentrations observed in other species under conditions of global tissue ischemia (Bayly *et al.,* 1983; McCoy *et al.,* 2011; Haas *et al.,* 2016). This finding adumbrates that the high Qt of these rhinoceros under etorphine might be lifesaving in the face of high VO_2_ and low CaO_2_. Because the high Qt is caused by increased f_H_, any therapy that reduces f_H_ (e.g. an α_2_ adrenoceptor agonist sedative like detomidine) without decreasing VO_2_ might exacerbate metabolic acidosis. The clinical implication of this is that co-administration of α_2_ adrenoceptor agonist sedatives that cause bradycardia, e.g. detomidine or medetomidine, might be contraindicated when etorphine is used. It is also important to note that much greater plasma lactate concentrations are reported in rhinoceros darted with etorphine during field capture, where exertion and other stressors might exacerbate tissue hypoxia and thus induce anaerobic metabolism (Haw *et al.,* 2015; de Lange *et al.,* 2017).

#### Cardiopulmonary Effects

##### Hypoxaemia

Etorphine immobilization consistently resulted in severe, persistent hypoxaemia (PaO_2_ ~ 27 mm Hg, CaO_2_ ~ 14 ml dl^−1^ at t30). Possible causes of the hypoxaemia include reduced FiO_2_, hypoventilation and increased pulmonary venous admixture. The atmospheric pressure where the observations were made (~740 mm Hg) meant that inspired oxygen pressure was ~155 mm Hg (FiO_2_ ≅ 21%), suggested that reduced FiO_2_ would not have made an important contribution to alveolar or arterial hypoxia. Minute ventilation, however, was significantly less than predicted allometrically, and PaCO_2_ was >80 mm Hg throughout immobilization, approaching 100 mm Hg in some rhinoceros. This confirmed that substantial alveolar hypoventilation was present during etorphine immobilization. Hypoventilation likely contributed to the hypoxaemia by increasing alveolar carbon dioxide, which displaced the other alveolar gases, including oxygen (Riley and Cournand, 1951). The observed Pa-aO_2_ values (>30 mm Hg) were much greater than values generally seen in resting, healthy mammals (<10 mm Hg), suggesting that venous admixture was present in the rhinoceros and that it made a substantial contribution to the hypoxaemia (Hopkins *et al.,* 1994, 1998). The data suggest possible causes of venous admixture in these rhinoceros. The values for Qt under etorphine were 65% greater than predicted allometrically, and the values for pulmonary vascular pressures exceeded those reported in resting horses and humans (Schmidt-Nielsen, 1991; Sinha *et al.,* 1996; Naeije and Chesler, 2012). Noradrenaline agonism of β_1_ adrenoceptors in the sinoatrial node could explain the increased f_H_, which increased pulmonary blood flow (i.e. right-sided Qt). The increased pulmonary blood flow could have reduced erythrocyte transit time through the pulmonary capillaries, shortening the duration of erythrocyte exposure to alveolar gas and decreasing the time for oxygen to diffuse across the alveolar-capillary membrane and bind to Hb, resulting in abnormally low capillary blood oxygen content (Riley and Cournand, 1951). Interstitial pulmonary oedema increases the diffusion path for oxygen across the alveolar-capillary membrane and can occur when fluid flux (J_v_) from the capillary lumen to the interstitial space increases (Levick and Michel, 2010). Clinically, the most common reasons for an increase in J_v_ are increased capillary hydrostatic pressure (Pc), damage to the alveolar-capillary membrane (which increases conductivity, K_f_, and decreases the Staverman reflection coefficient, σ), or decreased capillary oncotic pressure (Πc) (Starling, 1896; Murray, 2011). Although techniques for confirming the presence of interstitial pulmonary oedema were unavailable, if it had contributed to the hypoxemia, increased Pc or damage to the alveolar-capillary membrane would be the most likely mechanisms responsible for it. The mean pulmonary vascular pressures were so great in the etorphine-immobilized white rhinoceros that the rate of extravasation of water across the pulmonary capillaries would have increased and could have even caused stress failure of pulmonary capillaries, as has been described in horses during maximal exertion (exercise-induced pulmonary haemorrhage, or EIPH); this may explain unpublished reports of sanguineous froth observed at the nares of rhinoceros during immobilization with etorphine (West *et al.,* 1993). If the rate of water extravasation exceeded the lymphatic capacity to remove the water, interstitial pulmonary oedema would have occurred; such interstitial oedema would increase the barrier to diffusion of oxygen across the alveolar-capillary membrane and contribute to venous admixture (Starling, 1896; Riley and Cournand, 1951). Parts of the lung with lesser ventilation and/or greater perfusion also contribute to venous admixture; conventionally this is expressed as parts with ventilation:perfusion (V:Q) of <1 (Riley and Cournand, 1949). Electrical impedance tomography has demonstrated that the dependent lung is poorly ventilated in etorphine-immobilized, laterally recumbent white rhinoceros (Mosing *et al.,* 2020). Our data do not allow us to comment on the possible role of V:Q inequality in the venous admixture and hypoxemia observed in etorphine-immobilized rhinoceros. It is worth noting that the CῡO_2_ in etorphine-immobilized rhinoceros is ~5 ml dl^−1^; this is much less than considered normal in horses in which it is ordinarily ~15 ml dl^−1^ (Butler *et al.,* 1993). This would amplify the effects of all the above causes for venous admixture and therefore contribute to arterial hypoxemia (Riley *et al.,* 1951).

##### Hypoventilation

The metabolic and cardiopulmonary responses to etorphine described above can largely be attributed to those expected from sympathetic upregulation. On the other hand, ventilation was apparently downregulated; both f_R_ and Vebtps were less than predicted allometrically (Schmidt-Nielsen, 1991). This finding is consistent with the depressant effects of μ opioid agonists on breathing pattern generation by the medullary pre-Bötzinger and pontine parabrachial complexes that have been described in other mammals at doses equal to or greater than those used to produce analgesia (Lalley, 2006; Prkic *et al.,* 2012; Miller *et al.,* 2017; Saunders and Levitt, 2020). A consequent decrease in alveolar ventilation, along with hyper-metabolically increased carbon dioxide production, would account for the observed hypercapnia.

### Effects of Butorphanol in White Rhinoceros Immobilized With Etorphine

#### Plasma Catecholamine Concentrations

Butorphanol administration had no effect on mean plasma noradrenaline concentration; at t40 and t50, it still exceeded concentrations in the non-immobilized rhinoceros. Given that the half-life of noradrenaline is on the order of 2.5 min, this suggests that the butorphanol was not responsible for a substantial normalization of sympathetic activity (Silverberg *et al.,* 1978). Butorphanol is a partial agonist of μ opioid receptors and opposes full agonism of μ opioid receptors by pure agonists like etorphine (Garner *et al.,* 1997). Since μ opioid receptors are likely responsible for the plasma noradrenaline concentrations seen under etorphine (above), one might expect that butorphanol would substantially decrease plasma noradrenaline concentrations as it displaces etorphine from the μ opioid receptors. However, butorphanol is also an agonist of κ opioid receptors, and κ opioid receptor agonism is also capable of activating sympathetic pathways and increasing plasma adrenaline and noradrenaline concentrations, as documented after nalbuphine administration to healthy men (Chang *et al.,* 1981; Hoehe and Duka, 1993). Kappa opioid receptor agonism could explain why a substantial decrease in plasma noradrenaline concentration was not observed after butorphanol administration. Nevertheless, the effects of butorphanol on the metabolic and cardiopulmonary complications caused by etorphine were considerable and largely beneficial, which suggests a pathway independent of plasma noradrenaline concentration; however, our data do not allow us to exclude central sympatholytic effects.

#### Metabolic Effects

##### Muscle Tremors

Butorphanol reduced tremor score via an unknown mechanism. Electromyography in standing horses has demonstrated that butorphanol relaxes skeletal muscle; however, in isolated equine skeletal muscle in vitro, butorphanol had no effect on contraction, suggesting a central mechanism of muscle relaxation (Wooldridge *et al.,* 2002; Kruluc and Nemec, 2006). Butorphanol-induced muscle relaxation could explain the observed decrease in VO_2_.

#### Cardiopulmonary Effects

##### Hypoxaemia

Butorphanol significantly improved PaO_2_, increasing it from ~27 mm Hg at baseline to ~47–49 mm Hg. However, PaO_2_ is ~90–100 mm Hg in non-immobilized white rhinoceros habituated to arterial puncture; thus, butorphanol did not normalize PaO_2_ completely (Citino and Bush, 2007). Despite residual hypoxaemia, the increase in PaO_2_ following butorphanol administration raised CaO_2_ from ~15 to ~20 ml dl^−1^, a value considered normal in horses (Butler *et al.,* 1993). Given that [Hb] did not increase after butorphanol administration, the normalization of CaO_2_ while PaO_2_ remains subnormal is likely due to the characteristics of the ODC of the white rhinoceros, which has a P_50_ (oxygen partial pressure at which haemoglobin is 50% saturated) of 20.6 mm Hg, less than that of dogs, humans and horses (28.8, 26.6 and 23.8 mm Hg, respectively) (Clerbaux *et al.,* 1993; Reiners *et al.,* 2019; Shepherd *et al.,* 2019). A substitution of glutamic acid for glutamine at the β_2_ position of haemoglobin appears to drive the leftward ‘shift’ in the ODC in white rhinoceroses. This shift causes their haemoglobin to bind substantially more oxygen at PaO_2_ values in the 45–55 mm Hg range compared to other mammals (Mazur *et al.,* 1982).

Butorphanol could have improved PaO_2_ in a number of ways. Butorphanol decreased PaCO_2_; this is compatible with reduced alveolar carbon dioxide partial pressure permitting an increase in PaO_2_ and, in turn, increasing PaO_2_ (Fenn *et al.,* 1946; Riley and Cournand, 1949; Riley *et al.,* 1951). Butorphanol may have decreased the f_H_ via vagal stimulation; kappa agonists have induced bradycardia in rats (Gulati and Bhargava, 1988; Hassen and Broudy, 1988). This effect would decrease Qt and pulmonary vascular pressures, and therefore the hydrostatic pressure driving water into the extravascular space in the pulmonary bed (Starling, 1896). The decrease in Qt would also have increased pulmonary capillary transit time. The decrease in VO_2_ was associated with an increase in CῡO_2_ to values that approach those considered normal in horses (Butler *et al.,* 1993). In turn, the tendency to normalize CῡO_2_ would have reduced the influence of all the possible causes of pulmonary venous admixture (including reduced Hb transit time, interstitial oedema and V:Q < 1) (Riley *et al.,* 1951).

Despite CaO_2_ increasing substantially after butorphanol was administered, DO_2_ did not increase and, in fact, decreased significantly, requiring us to reject our hypothesis that butorphanol would increase DO_2_ in white rhinoceros under etorphine. The immediate mechanism behind this observation is that a substantial decrease in Qt was observed after butorphanol administration; this was largely driven by a decrease in f_H_, as SV did not change significantly. The decrease in Qt more than balanced the increase in CaO_2,_ causing DO_2_ to decrease.

The OER was not significantly improved by butorphanol. This finding can be explained by the proportionate decrease in both VO_2_ and DO_2_. Inspection of the data suggests that variability in the OER data might conceal a transient reduction that did not achieve statistical significance 3 min after butorphanol was administered (i.e. t40). Butorphanol did not produce a significant reduction in lactate, suggesting that anaerobic metabolism was unchanged by butorphanol. However, the increase in CῡO_2_ nevertheless suggests an improved oxygen supply-and-demand balance.

##### Hypoventilation

Three minutes after it was given, butorphanol transiently reduced PaCO_2_ due to a transient increase in f_R_. This effect is consistent with the butorphanol displacing the pure opioid agonist etorphine from μ receptors on neurons in the medulla and pons that are responsible for the rhythm generation of ventilation (Lalley, 2006; Prkic *et al.,* 2012).

## Limitations

The major study limitations are the small number of white rhinoceros and the lack of normal ranges for several physiological variables in non-immobilized white rhinoceros due to the challenges posed by invasive monitoring in conscious rhinoceros. However, the use of non-immobilized rhinoceros for establishing normal plasma catecholamine concentrations represents an important step forward in providing reference values for rhinoceros that will benefit future studies.

## Summary and Conclusions

In white rhinoceros, the ultra-potent opioid agonist, etorphine, causes sympathetic upregulation characterized by plasma noradrenaline concentrations approximately twice those measured in habituated, non-immobilized white rhinoceros; this sympathetic upregulation contributes to severe arterial hypoxaemia. The hypoxaemia is not only caused by hypoventilation but by increased venous admixture. Sympathetic upregulation, characterized by increased plasma noradrenaline concentration, could contribute to the venous admixture. Increased f_H_ due to β_1_ adrenoceptor agonism increased Qt and therefore pulmonary pressures. This effect could have produced venous admixture by decreasing erythrocyte transit time through the pulmonary capillaries and/or by increasing Pc sufficiently to cause pulmonary oedema. The high VO_2_ produced mixed venous hypoxaemia, which amplified the effects of pulmonary venous admixture. Despite sympathetic upregulation, etorphine depressed ventilation; this is in accord with respiratory depression ordinarily associated with μ opioid agonist activity in the brainstem.

When butorphanol, a partial μ opioid agonist and pure κ opioid agonist, was administered to white rhinoceros under etorphine, the ventilatory depression caused by etorphine was attenuated, consistent with partial agonism of, and displacement of etorphine from, μ opioid receptors in medullary and pontine centres responsible for control of breathing. Shortly after butorphanol administration, plasma adrenaline concentration was transiently greater than contemporary values for rhinoceros under etorphine alone; otherwise, the effects of butorphanol on plasma adrenaline, noradrenaline and dopamine concentrations were not significant. Plasma noradrenaline concentration remained much higher than expected, while Qt, pulmonary pressures and VO_2_ decreased from high values under etorphine alone (although they did not reach values considered normal). Butorphanol decreased Qt and pulmonary pressures by decreasing f_H_. The increase in PaO_2_ and CaO_2_ can be explained not only by the attenuation of ventilatory depression but by the decrease in Qt. However, the decrease in Qt offset the increased CaO_2_, this so that DO_2_ was actually reduced after butorphanol. Butorphanol reduced VO_2_, apparently by mitigating tremors. The effect of the decrease in VO_2_ was that PῡO_2_ and CῡO_2_ increased, thereby reducing the impact of venous admixture on CaO_2_. The partial normalization of the cardiopulmonary and metabolic values by butorphanol cannot be explained by a decrease in plasma catecholamine concentrations. Maintenance of increased plasma noradrenaline concentration could be a result of κ opioid agonism by butorphanol. Alternatively, butorphanol could decrease DO_2_ and VO_2_ via mechanisms independent of plasma noradrenaline concentration. Plasma catecholamine concentrations do not necessarily reflect neurotransmitter activity within the sympathetic pathways of the nervous system; therefore butorphanol could still have caused sympatholytic effects.

Future research should aim to mitigate the sympathetic upregulation induced by etorphine. Although butorphanol did not improve DO_2_, pending a practical alternative, its continued use in etorphine-immobilized white rhinoceros is recommended because it counteracts etorphine-induced hypermetabolism, thus reducing VO_2_ towards normal and improving the oxygen supply (DO_2_) and demand (VO_2_) balance.

## Supplementary Material

Web_Material_coaf009

## Data Availability

All relevant data and resources can be found within the article and its supplementary information.
