# Supplementary material for: Etorphine induces pathophysiology in immobilized white rhinoceros through sympathomimesis that is attenuated by butorphanol
Source: Conserv Physiol. 2025 Apr 4;13(1):coaf009. doi: 10.1093/conphys/coaf009 (PMC11974248; doi:10.1093/conphys/coaf009)
Supplement: Web_Material_coaf009 [file web_material_coaf009.docx]

SUPPLEMENTAL INFORMATION

Table S1. Information regarding the 11 non-immobilised white rhinoceros from which “control” venous samples were collected for catecholamine assay. The rhinoceros were subadult or adult orphans that were habituated for blood collection. Five years old is considered the onset of adulthood.

| Name | Age | Sex | Adrenaline (ng mL^-1^) | Noradrenaline  (ng mL^-1^) | Dopamine  (ng mL^-1^) |
| --- | --- | --- | --- | --- | --- |
| Yster | 3 years 5 months | M | 0.82 | 26.01 | 2.14 |
| Sophia | 5 years 4 months | F | 0.81 | 23.77 | 2.14 |
| Eli | 2 years 4 months | M | 0.82 | 22.49 | 2.14 |
| Blossom | 4 years 7 months | F | 0.82 | 24.40 | 2.16 |
| Ribbon | 4 years 10 months | F | 0.84 | 21.48 | 2.22 |
| Summer | 5 years 7 months | F | 0.83 | 26.39 | 2.15 |
| Arthur | 5 years 2 months | M | 0.81 | 24.77 | 2.13 |
| Thomas | 3 years 3 months | M | 0.81 | 26.78 | 2.15 |
| Ranger | 3 years 8 months | M | 0.84 | 11.28 | 2.20 |
| Freddie | 3 years 1 months | M | 0.81 | 27.65 | 2.13 |
| Anchor | 3 years 2 months | M | 0.82 | 28.06 | 2.13 |

Table S2. Standard doses of drugs used in white rhinoceros by Veterinary Wildlife Services (VWS) based on body weight; all rhinoceros used in this study fell into one of these categories.

| Rhinoceros weight (kg) | Etorphine  (mg) | Hyaluronidase (IU) | Butorphanol  (10x etorphine dose, mg) | Naltrexone  (20x etorphine dose, mg) |
| --- | --- | --- | --- | --- |
| 1,000-1,250 | 2.5 | 5,000 | 25 | 50 |
| 1,250-1,500 | 3.125 | 5,000 | 31.25 | 62.5 |

IU, International Units

Table S3. Subjective scoring system used to assess severity of tremors in etorphine-immobilised white rhinoceros (Buss et al., 2018).

| Score | Description |
| --- | --- |
| 1 | No visible tremors |
| 2 | Slight tremors – resulting in minor leg and foot movement |
| 3 | Mild tremors – resulting in minor shoulder, chest and severe leg and foot movement |
| 4 | Moderate tremors – resulting in severe shoulder, chest, leg and foot movement |
| 5 | Severe tremors – resulting in whole body and head movement |

Table S4. Equations (alphabetical) for calculated physiological variables in etorphine-immobilised white rhinoceros.

| Calculated Variable | Equation |
| --- | --- |
| CaO_2_ (mL dL^-1^) | ([Hb] × 1.39 × SaO_2_) + (PaO_2_ × 0.003) |
| CῡO_2_ (mL dL^-1^) | ([Hb] × 1.39 × SῡO_2_) + (PῡO_2_ × 0.003) |
| DO_2_ (mL min^-1^ kg^-1^) | CaO_2_ × Qt |
| FĒCO_2_ | PĒCO_2_ ÷ Pb |
| [Hb] (g dL^-1^) | (0.2528 × PCV) + 4.013 |
| OER (%) | VO_2_ ÷ DO_2_ |
| PaO_2_ (mm Hg) | FiO_2_ × [(P_B_ − P_WV_) – (PaCO_2_ ÷ 1)] |
| Pa-aO_2_ (mm Hg) | PaO_2_ − PaO_2_ |
| PVR (mm Hg × min L^-1^) | (mPAP – mPAOP) ÷ Qt |
| SV (mL beat^-1^ kg^-1^) | Qt ÷ f_H_ |
| VCO_2_ (mL min^-1^ kg^-1^) | (Vestpd × FĒCO_2_ ) − (Vi × FiCO_2_) |
| Vdphys (mL min^-1^ kg^-1^) | [(PaCO_2_ − PĒCO_2_) ÷ PaCO_2_] × Vt − 0.3† |
| VO_2_ (mL min^-1^ kg^-1^) | (Vi × FiO_2_) − (Vesptd × FĒO_2_)ǂ |
| Vestpd (mL min^-1^ kg^-1^) | (273 ÷ 310) × [(Pb − 47) ÷ 760] × Vebtps |
| Vt (mL breath^-1^ kg^-1^) | Vestpd ÷ f_R_ |

CaO_2_, arterial oxygen content; CῡO_2_, mixed venous oxygen content; DO_2_, oxygen delivery; FĒCO_2_, mixed expired carbon dioxide fraction; FĒO_2_, mixed expired oxygen fraction; f_H_, heart rate; FiCO_2_, inspired carbon dioxide fraction; FiO_2_, inspired oxygen fraction; [Hb], haemoglobin concentration; f_R_, respiratory rate; mPAP, mean pulmonary arterial pressure; mPAOP, mean pulmonary arterial occlusion pressure; OER, oxygen extraction ratio; PaCO_2_, arterial carbon dioxide partial pressure; PaO_2_, alveolar oxygen partial pressure; PaO_2_, arterial oxygen partial pressure; Pa-aO_2_, alveolar-arterial oxygen partial pressure difference; Pb, barometric pressure; PCV, packed cell volume; PĒCO_2_, mixed expired carbon dioxide partial pressure; PVR, pulmonary vascular resistance; PῡO_2_, mixed venous oxygen partial pressure; Pwv, saturated water vapour pressure; Qt, cardiac output; SaO_2_, arterial oxyhaemoglobin saturation; SV, stroke volume; SῡO_2_, mixed venous oxyhaemoglobin saturation; VCO_2_, carbon dioxide production; Vdphys, physiological dead space ventilation; Vebtps, minute ventilation, body temperature and pressure standard; Vestpd, minute ventilation, standard temperature and pressure dry; Vi, inspired volume; VO_2_, oxygen consumption; Vt, tidal volume.

†0.3 L was subtracted to account for the volume of the tubes external to the nares.

^ǂ^Vi was not necessarily equivalent to Ve because VO_2_ did not necessarily equal VCO_2_ [i.e., the respiratory quotient (RQ) was not necessarily = 1]. Therefore, the Haldane transformation was used to determine Vi (Poole and Whipp, 1988).

Table S5. Physiological variables measured or calculated in healthy, boma-habituated, sub-adult, male white rhinoceros (n = 6), assigned to two treatments administered once each in random order: etorphine-saline (ES) and etorphine-butorphanol (EB). Data were collected 30, 40, and 50 min after the rhinoceros became recumbent (t30, t40, and t50, respectively); either 0.9% saline (treatment ES) or butorphanol (treatment EB) was injected IV at t37.

|  | t30 | | t40 | | t50 | |
| --- | --- | --- | --- | --- | --- | --- |
|  | mean | SD | mean | SD | mean | SD |
| **Catecholamines (Figure 1)** | | | | | | |
| **Adrenaline (ng mL^-1^)** |  |  |  |  |  |  |
| **ES** | **0.73** | **0.19** | **0.41** | **0.26** | **0.89** | **0.33** |
| **EB** | **0.87** | **0.18** | **0.69** | **0.21** | **0.78** | **0.09** |
| **Noradrenaline (ng mL^-1^)** |  |  |  |  |  |  |
| **ES** | **49.25** | **10.44** | **39.09** | **14.07** | **39.13** | **3.7** |
| **EB** | **38.35*** | **2.77** | **35.8** | **8.41** | **39.94** | **6.38** |
| **Dopamine (ng mL^-1^)** |  |  |  |  |  |  |
| **ES** | **1.28** | **0.03** | **1.29** | **0.03** | **1.27** | **0.03** |
| **EB** | **1.28** | **0.03** | **1.27** | **0.02** | **1.30** | **0.02** |
| **Metabolic Variables (Figure 2)** | | | | | | |
| **VO_2_ (mL min^-1^ kg^-1^)** |  |  |  |  |  |  |
| **ES** | **4.8** | **0.9** | **4.5†** | **0.6** | **5.4†** | **0.6** |
| **EB** | **4.9** | **1.0** | **2.9†*** | **0.4** | **3.9†*** | **0.5** |
| T_PA_ (°C) |  |  |  |  |  |  |
| ES | 38.4 | 0.6 | 38.3 | 0.6 | 38.2 | 0.6 |
| EB | 38.0 | 0.7 | 37.2†∞ | 0.7 | 37.5†∞ | 0.5 |
| T_R_ (°C) |  |  |  |  |  |  |
| ES | 38.5 | 0.4 | 38.6 | 0.6 | 38.6 | 0.6 |
| EB | 38.2 | 0.7 | 38.2 | 0.7 | 37.7†∞ | 0.8 |
| Tremor score |  |  |  |  |  |  |
| ES | 2.8 | 0.4 | 2.0† | 0.0 | 2.0† | 0.0 |
| EB | 2.7 | 0.5 | 1.5† | 0.5 | 1.3†∞ | 0.5 |
| Lactate^a^ (mmol L^-1^) |  |  |  |  |  |  |
| ES | 2.1 | 1.8 | 1.8 | 1.5 | 1.6 | 1.3 |
| EB | 1.7 | 1.2 | 1.6 | 1.0 | 1.3 | 0.7 |
| OER (%) |  |  |  |  |  |  |
| ES | 44.5 | 8.8 | 40.7 | 11.2 | 49.2 | 9.2 |
| EB | 44.8 | 7.4 | 34.3 | 10.7 | 53.3 | 15.2 |
| VCO_2_ (mL min^-1^ kg^-1^) |  |  |  |  |  |  |
| ES | 4.2 | 1.1 | 3.9 | 0.7 | 4.6 | 0.5 |
| EB | 4.0 | 1.0 | 4.6 | 1.2 | 3.9ǂ | 0.5 |
| **Cardiovascular Variables (Figures 3 and 4)** | | | | | | |
| **Qt (mL min^-1^ kg^-1^)** |  |  |  |  |  |  |
| **ES** | **81** | **26** | **80** | **22** | **73** | **13** |
| **EB** | **77** | **21** | **45†*** | **14** | **40†*** | **11** |
| f_H_ (beats min^-1^) |  |  |  |  |  |  |
| ES | 121 | 21 | 121 | 16 | 116 | 21 |
| EB | 121 | 17 | 83†∞ | 14 | 71†∞ | 12 |
| SV (mL beat^-1^ kg^-1^) |  |  |  |  |  |  |
| ES | 0.7 | 0.1 | 0.7 | 0.2 | 0.6 | 0.0 |
| EB | 0.6 | 0.2 | 0.6 | 0.3 | 0.6 | 0.2 |
| mSAP (mmHg) |  |  |  |  |  |  |
| ES | 150 | 25 | 145 | 20 | 145 | 17 |
| EB | 140 | 23 | 110ǂ∞ | 30 | 146 | 51 |
| **PaO_2_ (mmHg)** |  |  |  |  |  |  |
| **ES** | **26.5** | **3.4** | **27.8** | **4.1** | **28.9** | **3.4** |
| **EB** | **27.2** | **4.3** | **48.8†*** | **3.5** | **46.8†*** | **3.0** |
| CaO_2_ (mL dL^-1^) |  |  |  |  |  |  |
| ES | 14.3 | 2.7 | 14.6 | 2.3 | 15.5 | 2.1 |
| EB | 14.7 | 2.7 | 19.8†* | 1.2 | 19.1†* | 1.0 |
| **PῡO_2_ (mmHg)** |  |  |  |  |  |  |
| **ES** | **18.0** | **3.7** | **20.0** | **3.9** | **21.7†** | **3.4** |
| **EB** | **19.5** | **4.6** | **34.4†*** | **2.1** | **32.3†*** | **3.5** |
| **DO_2_ (mL min^-1^ kg^-1^)** |  |  |  |  |  |  |
| **ES** | **11.1** | **1.9** | **11.5** | **2.9** | **11.2** | **1.6** |
| **EB** | **11.0** | **2.2** | **9.0*** | **2.8** | **7.7†*** | **2.0** |
| CῡO_2_ (mL dL^-1^) |  |  |  |  |  |  |
| ES | 9.9 | 2.4 | 10.7 | 2.3 | 11.6ǂ | 1.8 |
| EB | 11.0 | 3.0 | 17.7†* | 1.6 | 16.3†* | 1.8 |
| [Hb] (g dL^-1^) |  |  |  |  |  |  |
| ES | 15.6 | 0.4 | 15.1 | 0.5 | 15.1 | 0.4 |
| EB | 16.0 | 0.9 | 15.7 | 1.1 | 15.0† | 0.9 |
| **Pulmonary Variables (Figures 5 and 6)** | | | | | | |
| PaCO_2_ (mmHg) |  |  |  |  |  |  |
| ES | 86.3 | 6.5 | 87.8 | 8.5 | 82.9 | 5.8 |
| EB | 87.8 | 6.1 | 65.1†* | 9.5 | 68.7†∞ | 9.0 |
| f_R_ (breaths min^-1^) |  |  |  |  |  |  |
| ES | 5.6 | 2.3 | 6.3 | 2.6 | 7.4 | 2.2 |
| EB | 6.0 | 2.1 | 10.9†* | 1.9 | 10.0†∞ | 1.2 |
| Pa-aO_2_ (mmHg) |  |  |  |  |  |  |
| ES | 31.1 | 5.5 | 29.5 | 8.9 | 33.4 | 6.8 |
| EB | 28.2 | 7.7 | 31.0 | 10.9 | 27.0 | 10.9 |
| Vdphys (mL min^-1^ kg^-1^) |  |  |  |  |  |  |
| ES | 47.3 | 10.9 | 48.7 | 11.1 | 48.2 | 9.1 |
| EB | 45.5 | 10.8 | 51.6 | 15.2 | 47.7 | 6.2 |
| Vebtps (mL min^-1^ kg^-1^) |  |  |  |  |  |  |
| ES | 100.1 | 19.9 | 95.4 | 15.1 | 90.9 | 10.1 |
| EB | 96.6 | 20.5 | 112.0ǂ∞ | 29.0 | 96.0 | 11.4 |
| Vt (mL breath^-1^ kg^-1^) |  |  |  |  |  |  |
| ES | 19.6 | 7.0 | 16.7 | 5.1 | 14.1ǂ | 3.6 |
| EB | 17.9 | 7.2 | 10.1ǂ∞ | 1.0 | 9.7ǂ | 0.7 |
|  | | | | | | |
| **mPAP (mmHg)** |  |  |  |  |  |  |
| **ES** | **59** | **8** | **56** | **10** | **56** | **8** |
| **EB** | **62** | **12** | **48†** | **7** | **45†*** | **7** |
| **mPAOP (mmHg)** |  |  |  |  |  |  |
| **ES** | **37** | **9** | **34** | **8** | **34** | **6** |
| **EB** | **33** | **8** | **27†*** | **7** | **28†** | **6** |
| PVR (mmHg × min L^-1^) |  |  |  |  |  |  |
| ES | 0.26 | 0.07 | 0.26 | 0.10 | 0.27 | 0.05 |
| EB | 0.37 | 0.20 | 0.42∞ | 0.08 | 0.42 | 0.25 |

Primary outcomes are indicated in bold. †Significantly different from value in same treatment at t30, after Bonferroni correction. *Significantly different from contemporary ES value. ǂSignificantly different from value in same treatment at t30 before Bonferroni correction. ∞Significantly different from contemporary ES value before Bonferroni correction. NB, differences that lost significance after Bonferroni correction were not considered significant for the purposes of discussion in this paper.

SD, standard deviation; VO_2_, oxygen consumption; T_PA_, pulmonary artery temperature; T_R_, rectal temperature; OER, oxygen extraction ratio; VCO_2_, carbon dioxide production; Qt, cardiac output; f_H_, heart rate; SV, stroke volume; mSAP, mean systemic arterial pressure; PaO_2_, arterial oxygen partial pressure; CaO_2_, arterial oxygen content; PῡO_2_, mixed venous oxygen partial pressure; DO_2_, oxygen delivery; CῡO_2_, mixed venous oxygen content; [Hb], haemoglobin concentration; PaCO_2_, arterial carbon dioxide partial pressure; f_R_, respiratory rate; Pa-aO_2_, alveolar-arterial oxygen partial pressure difference; Vdphys, physiological dead space ventilation; Vebtps, minute ventilation, body temperature and pressure saturated with water vapour; Vt, tidal volume; mPAP, mean pulmonary arterial pressure; mPAOP, pulmonary arterial occlusion pressure; PVR, pulmonary vascular resistance.

^a^Mixed venous lactate concentration.

Dataset S1. Details of the liquid-chromatography-mass spectrometry (LC-MS) technique used to measure plasma catecolamine concentrations in the white rhincoeros.

**Sample preparation**

Plasma samples were removed from the -80 °C refrigerator and thawed on ice before sample preparation. In a 1.5 mL micro-Eppendorf test tube, 100 µL of plasma or standard was combined with 250 µL of the preparation solution. This preparation solution consisted of 250 ng mL^-1^ of the internal standard 5-hydroxy-Nω-methyltryptamine (5-HMT) and methanol, acetonitrile, acetone, and formic acid in a 50:30:20:0.1 ratio (below).

The tube was vortexed for 10 seconds and left on ice for 30 minutes to allow precipitation of proteins. The sample was centrifuged at 20,817 g for 30 minutes at 4 ºC. After centrifuging, the supernatant was transferred to a glass insert in an amber sample vial, that was placed in the autosampler of the LC-MS for analysis. The results were reported in ng mL^-1^.

**Instrumentation**

A 6470 Triple Quad LC-MS was connected to a 1200 binary pump and autosampler controlled by MassHunter software (Agilent Technologies, Inc., Santa Clara, USA). The column was an Accucore^TM^-150-Amide-Hilic (250 x 2.1 mm, 2.6 µm, Thermo Fisher Scientific, Inc., Waltham, MA, USA).

**Standard solutions**

Approximately 1 mg of each analyte was dissolved separately in 10 mL of a 10% methanol solution in amber volumetric flasks. From the stock solutions of each analyte, a combined serial dilution series consisting of six to seven concentrations were prepared to construct a standard calibration curve and determine the linear range of each metabolite.

**Internal standard solution**

A stock solution of the internal standard, 5-HMT, was prepared at a concentration of 200 ng mL^-1^ using a solvent mixture of 0.1% (*v/v*) formic acid and 1:1 methanol:acetonitrile. Subsequently, a working internal standard solution with a final concentration of 250 ng mL^-1^ (above) was prepared from the stock solution using the same solvent mixture. This working solution was also used for the preparation of the different biological sample matrices and standards.

**Mobile phase**

A gradient mobile phase consisting of (A) 0.1% formic acid and HPLC grade water and (B) 0.1% formic acid and acetonitrile was prepared. Table indicates how the gradient elution of the mobile phase was applied. Three minutes (post-time) was allowed between (A) and (B) for the starting condition of the mobile phase mixture to reset.

Table S6. Mobile phase gradient setup.

| Step | Time (minutes) | A (%)  HPLC water/ 0.1% FA | B (%)  ACN/ 0.1% FA |
| --- | --- | --- | --- |
| 1 | Start condition 0 | 95.0 | 5.0 |
| 2 | 3.0 | 95.0 | 5.0 |
| 3 | 4.3 | 0.0 | 100.0 |
| 4 | 12.0 | 0.0 | 100.0 |
| 5 | 14.0 | 95.0 | 5.0 |
| 6 | 15.0 | 95.0 | 5.0 |
| 7 | Post-time 3 | 95.0 | 5.0 |

HPLC, high-performance liquid chromatography; FA, formic acid; ACN, acetonitrile.

**Multiple reaction monitoring optimisation**

A 1 mg mL^-1^ solution of each analyte was used to optimise ionisation and to determine the optimum settings for the detection product ions. Table S7. represents the optimum mass spectrometer settings for each metabolite precursor to qualify and quantify product ions.

Table S7. Optimum instrument settings for the identification and quantification of product ions.

| LC instrument settings | | | | | | | | |
| --- | --- | --- | --- | --- | --- | --- | --- | --- |
| Flow rate | | | | 0.3 mL min^-1^ | | | | |
| Injection volume | | | | 5 µL | | | | |
| Run time | | | | 18 min (15 min plus 3 min post-time) | | | | |
| Mass spectrometer settings | | | | | | | | |
| Source parameter | | | | Positive value | | | | |
| Gas temperature | | | | 350 °C | | | | |
| Gas flow | | | | 13 L min^-1^ | | | | |
| Nebulizer | | | | 60 | | | | |
| Capillary voltage | | | | 4000 V | | | | |
| Analyte setup | | | | | | | | |
| Analyte | Transition (m/z) | Dwell  (ms) | Fragmentor  (V) | | Collision energy  (V) | Polarity | Scan | RRT (min) |
| Dopamine1 | 154.1 to 137.0 | 100 | 60 | | 9 | Positive | MRM | ± 5.000 |
| Dopamine2 | 154.1 to 65.1 |  | 60 | | 37 |  |  | ± 5.000 |
| Noradrenaline1 | 170.1 to 152.0 |  | 60 | | 4 |  |  | ± 7.600 |
| Noradrenaline2 | 170.1 to 107.0 |  | 60 | | 20 |  |  | ± 7.600 |
| Adrenaline1 | 184.1 to 166.0 |  | 60 | | 8 |  |  | ± 5.900 |
| Adrenaline2 | 184.1 to 77.1 |  | 60 | | 48 |  |  | ± 5.900 |
| 5-HMT1 | 191.1 to 160.0 |  | 60 | | 13 |  |  | ± 4.300 |
| 5-HMT2 | 191.1 to 77.0 |  | 60 | | 69 |  |  | ± 4.300 |

5-HMT, 5-hydroxy-Nω-methyltryptamine; MRM, multiple reaction monitoring; RRT, relative retention time.

The calibration curves constructed were evaluated by means of interpreting respective linear regression values. Linearity was excellent over the respective calibration ranges (Table S8), with the corresponding coefficient of determination (R^2^) values consistently greater than 0.9999. All calibration curves and linearity were processed using GraphPad Prism 8 (GraphPad Software Inc., San Diego, CA, USA).

Table S8. Linear regression equation and coefficient of determination (R^2^).

| Analyte | Concentration range | Linear regression equation | R^2^ | LOQ  (ng mL^-1^) | LOD  (ng mL^-1^) |
| --- | --- | --- | --- | --- | --- |
| Dopamine | 1.953125; 3.9062; 7.8125; 15.625  31.25; 62.5  125; 250 ng mL^-1^ | y = (5463 × x) - 10015 | 0.9993 | 1.00 | 0.50 |
| Noradrenaline | 1.953125; 3.9062; 7.8125; 15.625  31.25; 62.5  125; 250 ng mL^-1^ | y = (1568 × x) - 4266 | 0.9984 | 1.00 | 0.50 |
| Adrenaline | 1.953125; 3.9062; 7.8125; 15.625  31.25; 62.5  125; 250 ng mL^-1^ | y = (8866 × x) - 6795 | 0.9979 | 0.50 | 0.25 |

LOQ, limit of quantification; LOD, limit of detection.
